# Context and Crowding in Perceptual Learning on a Peripheral Contrast Discrimination Task: Context-Specificity in Contrast Learning

**DOI:** 10.1371/journal.pone.0063278

**Published:** 2013-05-16

**Authors:** Nisha S. Yeotikar, Sieu K. Khuu, Lisa J. Asper, Catherine M. Suttle

**Affiliations:** School of Optometry & Vision Science, University of New South Wales, Sydney, Australia; Ecole Polytechnique Federale de Lausanne, Switzerland

## Abstract

Perceptual learning is an improvement in sensitivity due to practice on a sensory task and is generally specific to the trained stimuli and/or tasks. The present study investigated the effect of stimulus configuration and crowding on perceptual learning in contrast discrimination in peripheral vision, and the effect of perceptual training on crowding in this task. 29 normally-sighted observers were trained to discriminate Gabor stimuli presented at 9° eccentricity with either identical or orthogonally oriented flankers with respect to the target (ISO and CROSS, respectively), or on an isolated target (CONTROL). Contrast discrimination thresholds were measured at various eccentricities and target-flanker separations before and after training in order to determine any learning transfer to untrained stimulus parameters. Perceptual learning was observed in all three training stimuli; however, greater improvement was obtained with training on ISO-oriented stimuli compared to CROSS-oriented and unflanked stimuli. This learning did not transfer to untrained stimulus configurations, eccentricities or target-flanker separations. A characteristic crowding effect was observed increasing with viewing eccentricity and decreasing with target-flanker separation before and after training in both configurations. The magnitude of crowding was reduced only at the trained eccentricity and target-flanker separation; therefore, learning for contrast discrimination and for crowding in the present study was configuration and location specific. Our findings suggest that stimulus configuration plays an important role in the magnitude of perceptual learning in contrast discrimination and suggest context-specificity in learning.

## Introduction

Perceptual learning refers to improvement in sensitivity brought about through practice on sensory (perceptual) tasks. Visual perceptual training has been shown to improve various visual functions such as Vernier acuity [1], [2], contrast sensitivity [3]–[5], letter identification [6]–[8], orientation discrimination [9], [10], motion discrimination [11], [12], and face recognition [13]. The extent of perceptual learning has been found to depend on stimulus and task complexity. For example, tasks requiring discrimination on the basis of both spatial frequency and orientation of the stimulus yield more learning (greater enhancement) than those requiring discrimination of spatial frequency only [14].

A common characteristic of perceptual learning is specificity (failure to transfer to untrained parameters or locations) to the training stimulus and task [15]–[17]. Specificity is thought to reflect early cortical stage involvement in the learning process, which occurs in tasks such as orientation or contrast judgements based on one parameter of the target, rather than more complex tasks based on more than one parameter, such as identification [18]–[20]. According to the reverse hierarchy theory of perceptual learning, specificity is linked to task difficulty in a different way (on the basis of factors such as stimulus presentation, position uncertainty or target eccentricity), with more difficult tasks yielding more specific, lower level learning than easier tasks [21].

Stimulus complexity is dependent on parameters of the target and any contextual features (e.g. flankers) surrounding the target that may elicit suppressive or facilitatory interactions [22], and perceptually may enhance or reduce visibility due to factors such as ‘pop-out’ and crowding. Spatial context (e.g. lateral flankers) is generally considered responsible for *lateral masking*, which has some similarities, but is distinct from *crowding* [23], [24]. Crowding is reduced visibility due to surrounding elements, and is found in a range of tasks including letter identification (requiring detection, discrimination and identification of the letter; e.g. Chung, Levi, & Legge, 2001) [25], Vernier acuity and contrast discrimination; however, most previous findings show that crowding is negligible in tasks requiring detection (e.g. Saarinen & Levi, 1995, Levi and Carney, 2011, but see also Poder, 2008) [22], [23], [26]. Discrimination of a crowded target involves not only detection and discrimination of the target but also discrimination of the target from the surrounding features, and this additional requirement suggests that it occurs at a stage beyond simple detection (e.g. Levi, 2008) [27].

The crowding effect can be reduced by perceptual training, as has been shown in a letter identification task by training on the same task [7], [8], [28], [29] or on a contrast detection task [30]. The latter finding is interesting because it suggests that a task that is subject to crowding (letter identification) can be modified by training on a task that is widely found not to be subject to crowding (detection). These studies on the effect of perceptual training were carried out in the periphery, where crowding is strongest in the normal visual system, and crowding has been based on letter identification. Maniglia et al. (2011) found that improved contrast detection thresholds for horizontal Gabors flanked by iso-oriented Gabors did not transfer to identical horizontal Gabor target detection with orthogonal (vertical) flankers. This finding suggests that there was no learning following exposure to the target per se, and that the learning was context-specific, related to exposure to the target plus flankers. While the role of context in foveal perceptual learning has been controversial [31], [32], “context” may add to the task difficulty by inducing crowding and this may affect learning [33], [34].

The aims of the present study were to investigate whether context (in the form of flankers adjacent to the target) that may yield crowding facilitates perceptual learning and its transfer; and to investigate whether perceptual training affects crowding. A peripheral contrast discrimination task was employed for training on stimuli with and without flankers. We hypothesised that learning would be greater and less specific after training on flanked than on unflanked target stimuli, and that the perceptual training would reduce crowding in contrast discrimination. We found the learning to be specific to the trained location, target-flanker separation and to the stimulus configuration, with no transfer to untrained conditions, referred to here as context-specific learning. Crowding reduction was also specific to the trained conditions, and did not occur in any untrained conditions. Our findings support the idea that the visual system learns to discriminate the whole stimulus, including target plus flankers, and extend previous findings in spatial localisation [35], contrast detection [30] and letter identification [36] to contrast discrimination.

## Materials and Methods

All research investigations in this study have been conducted according to the principles expressed in the Declaration of Helsinki.

### 1. Observers

29 normally-sighted observers (age range 20–60 years, 18 females) participated in this study. Initial vision screening included examination for best corrected visual acuity, oculomotor balance, suppression (Worth four-dot test), and stereopsis (Randot stereo test). Only subjects within the normal limits on all tests were included; they also had a history of normal ocular and systemic health. Twenty-two observers were inexperienced in psychophysical experiments and seven were experienced observers. Four of these were naïve to the purpose of the study, and three were authors. The research was approved by the University of New South Wales Human Research Ethics Committee and written informed consent was obtained from each observer. All observers participated in pre-training, training and post-training tests.

### 2. Apparatus & Stimuli

Visual stimuli were generated using MATLAB software (version 7.2) and Psychtoolbox and were displayed on a 22″ (20″ viewable image size) flat profile, gamma corrected Mitsubishi Diamond Pro 2070^SB^ CRT monitor. The space-averaged screen luminance was 30.4 cd/m^2^ in an otherwise dark room.

Stimuli (target, reference and flankers) were Gabor patches consisting of a sinusoidal grating (spatial frequency (SF) of 6 cycles/degree) multiplied by a Gaussian envelope with a standard deviation (σ) of 0.16° (σ = ^1^/_SF_). The target stimulus consisted of a pedestal at 50% Michelson contrast and a superimposed test stimulus, which were identical in all parameters except contrast. The reference stimulus was identical to the pedestal, while the target contrast was always higher than the pedestal and varied during an experimental session. Thus, the test contrast was the difference between the target and the pedestal contrast. The target, pedestal and reference Gabors were of horizontal orientation.

The target and reference stimuli were flanked by six Gabors (flankers), three on each side, arranged laterally. Flanker contrast was always 70%. The centre-to-centre separation between any two Gabor stimuli was 4σ units (0.64°) in training, while the separation between the target/pedestal and flankers was varied in pre- and post-training tests (*target-flanker separation* paradigm; see below). A fixation point (0.48°) was located at the centre of the monitor throughout the experiments (except when the stimulus was presented centrally). The target/pedestal and reference stimuli were presented at equal eccentricity of 9° in training, however, the eccentricity was varied in pre- and post-training (*viewing eccentricity* paradigm).

**Figure 1:**
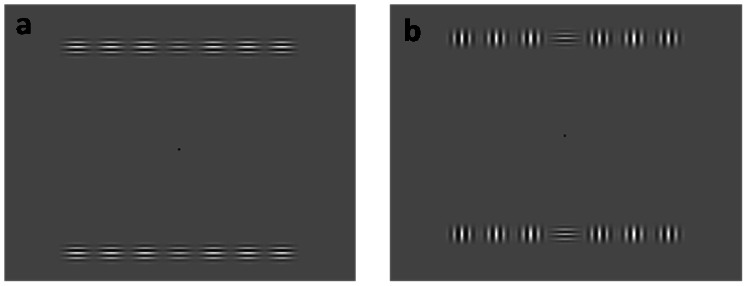
Stimulus configurations employed in the study; the central Gabors (either below or above fixation) are target and reference stimuli, while the three Gabors on each side of the central stimuli are flankers. Stimuli in this figure are located at 9° eccentricity and the target-flanker separation is 0.64°. Figure (a) and (b) represent Iso and Cross configuration respectively. Note that the size of the Gabor patch is increased here for the purpose of illustration.

Two basic stimulus configurations were used: Iso (horizontally oriented flankers, identical to the target/pedestal; Figure 1a) and Cross (vertically oriented flankers, orthogonal to the target/flankers; Figure 1b). In addition, an “isolated-target” (Control) condition was employed at all eccentricities. A detailed description of stimulus conditions in the two paradigms (viewing eccentricity and target-flanker separation) is given below.

### 3. Procedure

Observers viewed the stimuli from a distance of 75 cm using their non-dominant eye (based on sighting [37]), with the fellow eye covered with a translucent occluder.

Contrast discrimination thresholds were measured using a spatial two-alternative forced choice (2AFC) paradigm. Observers were simultaneously presented (250 ms duration) with both target and reference stimuli, each located randomly either above or below fixation. Spatial noise (random dots) with a central fixation point was presented initially and between trials to eliminate any afterimages. At “0°” eccentricity, the stimuli were only slightly eccentric (0.32°) and the fixation point was not visible during the stimulus presentation since the edge-edge separation of the target and reference stimuli was 0°. To ensure fixation, the point was presented prior to each trial (in the spatial noise) and then disappeared just before the stimulus presentation. The task was to indicate which stimulus (above or below fixation) appeared to be of higher contrast while maintaining central fixation. The stimulus presentation was accompanied by an auditory tone to reduce temporal uncertainty, and auditory feedback was provided after each correct response.

An adaptive random double staircase procedure with two-down and one-up (2/1) rule was used to obtain contrast discrimination thresholds (CDT). The first reversal on each staircase was excluded. The CDT was calculated as the mean of contrast levels at 10 reversals.

### 4. Study Design

Pre-training tests were completed in two sessions conducted on the same day. Each session was 30–40 minutes duration and included CDT measurement in one of the two paradigms. The order of these paradigms was randomised in pre- and post-training sessions and among the observers.

Training consisted of CDT measurement in 70 blocks in total (10 blocks per day) for each observer in one of the three stimulus conditions as follows: Group I: **ISO-9** (n = 10), Group II: **CROSS-9** (n = 10), and Group III: **CONTROL-9** (n = 9). The first term in these conditions indicates stimulus configuration or control, while the number indicates the eccentricity (9 degrees above or below fixation). Each training session was approximately 50 minutes duration, with each block consisting of about 70 trials. Therefore, each subject practiced around 5000 trials in total in seven sessions scheduled on seven different days. All observers completed the study within a period of 7–12 days.

The post-training sessions were conducted on the last day of the training and were identical to the pre-training sessions. Each subject took a break of at least an hour between the last training and first post-training session on that day to avoid any fatigue effects.

The two paradigms employed in pre- and post-training are described below:

#### 4.1. Paradigm I: viewing eccentricity

This paradigm included measurement of CDT at three eccentricities (0°, 4.5° and 9°) across the two configurations and in the isolated-target condition. The targets and flankers were located at the closest centre-centre separation in this paradigm (0.64°), which was employed to determine any transfer of learning to untrained eccentricities and stimulus configuration in terms of CDTs and the crowding effect.

#### 4.2. Paradigm II: Target-flanker (TF) separation

This paradigm included measurement of CDT at four TF separations (0.64°, 1.28°, 2.56°, and 5.12°) across the two configurations. The separations were calculated by multiplying the smallest centre-centre separation between the target (or reference) and flanking Gabor stimuli (0.64°) by 1, 2, 4 and 8. The target, flankers and reference were located at 9° in this paradigm. The paradigm was used to determine any transfer of learning to untrained TF separations and stimulus configuration in terms of CDTs and the crowding effect.

### 5. Data Analyses

All statistical analyses were conducted using Graphpad Prism (version 6). The data were considered in three parts for the purpose of analysis: (i) data collected during training (ii) pre- and post-training CDTs, and (iii) pre- and post-training relative CDTs (see below).

**Figure 2:**
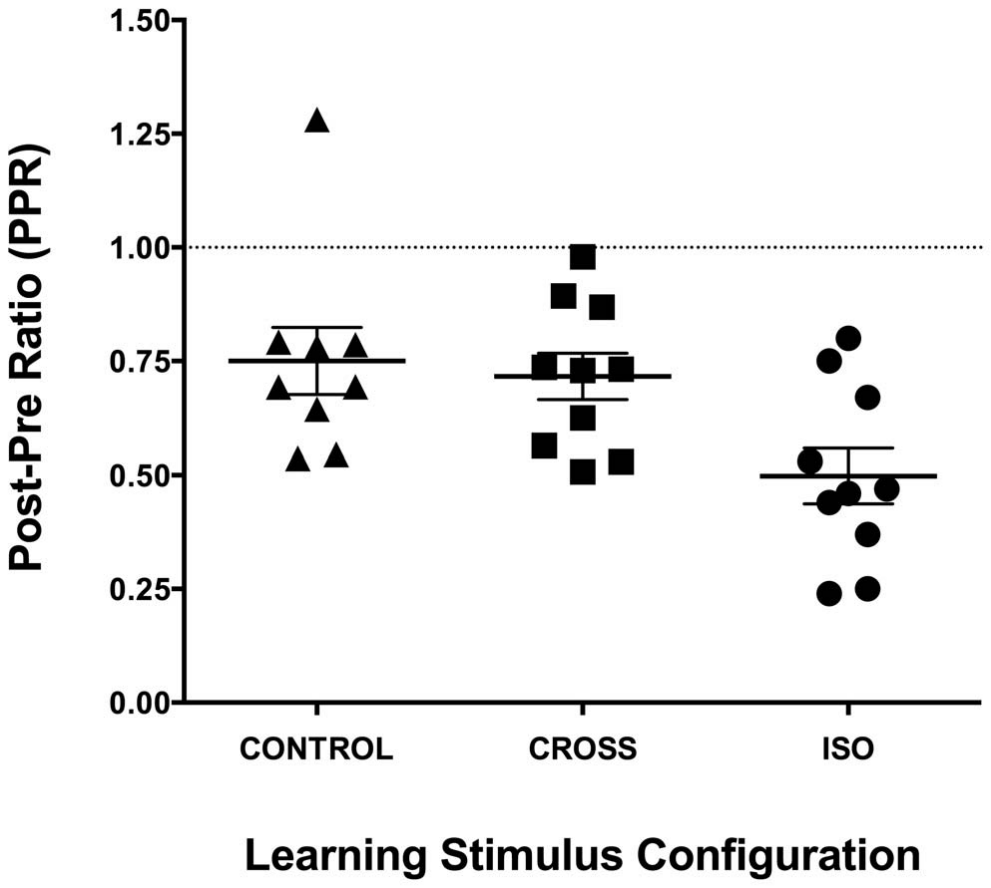
Post/pre contrast discrimination threshold ratios for the three training stimulus configurations (indicated by different symbols). The mean PPR for each group (solid lines; error bars signify standard error of the mean) is also shown in the Figure. The horizontal dashed line is indicative of no learning.

We looked for perceptual learning within individuals by calculating the ratio between post- and pre-training CDTs (post/pre ratio). While previous studies have shown overall reduction in thresholds during perceptual training, their data show considerable variation across sessions (e.g. Figure 5A in Fahle and Henke-Fahle, 1996; Figures 2 and 3 in Beard et al., 1995; Figure 2 in Sowden et al., 2002) [17], [38], [39]. A measure of post/pre training threshold ratio is subject to this variation. In an attempt to minimise the effects of variation, post and pre-training thresholds (within the training session only) were calculated as the mean of the last two and first two threshold values respectively in an observer’s training (Note that the post and pre-training thresholds used in this calculation are different from the thresholds measured separately in the pre- and post-training sessions.). Thresholds in the pre- and post-training sessions were used to measure the crowding effect in terms of relative thresholds, calculated as a ratio of CDT in the flanked to the unflanked (isolated-target) condition at both pre- and post-training. Thus, statistically significant elevation in relative threshold above 1.0 indicated crowding.

**Figure 3:**
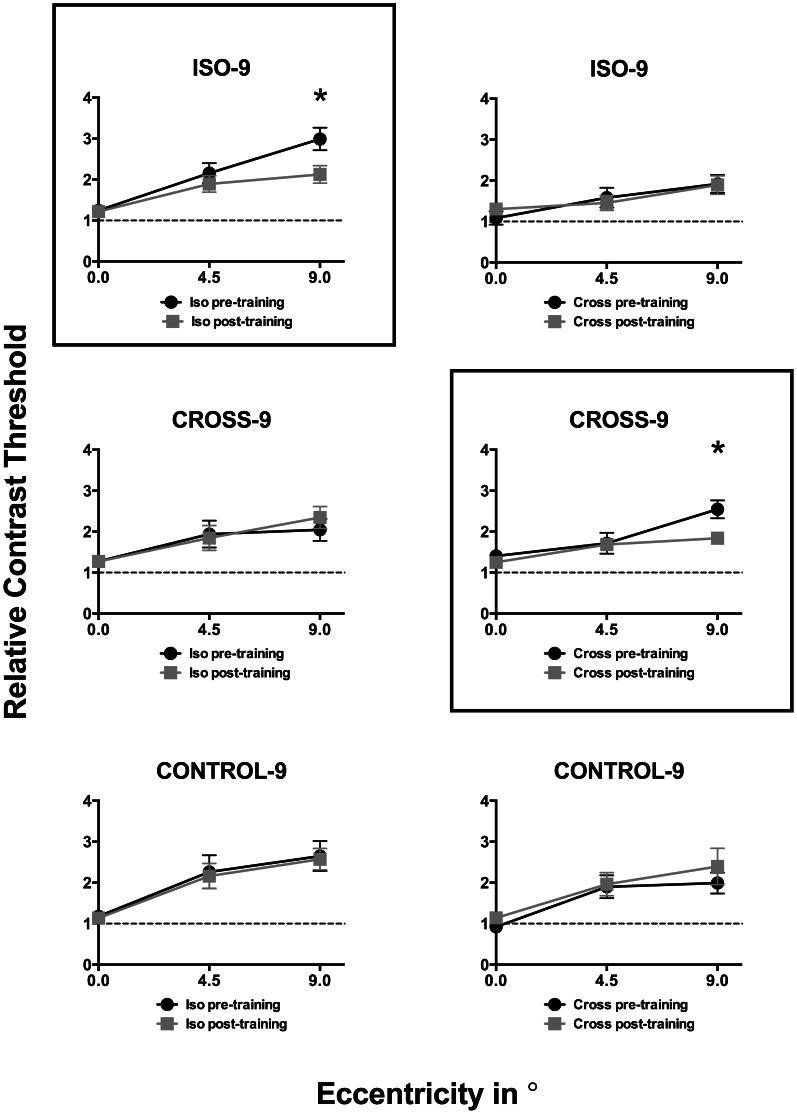
Relative contrast discrimination thresholds plotted as a function of eccentricity of the target stimulus. Data for pre- and post-training thresholds are represented by black circles and gray squares respectively. Error bars signify standard error of the mean. The different panels in Figure 3 depict data for the different training and test stimulus configurations grouped so that the rows indicate different training configurations, while columns indicate different test configurations. Framed panels indicate the training and test configurations in which a significant learning effect was observed; asterisks denote the condition in which the difference between pre- and post- contrast thresholds was statistically significant.

Note that the isolated-target condition is referred to as the “Control” condition in the Results and Discussion sections below. The specific statistical tests are mentioned along with the results in the following section.

## Results

Perceptual learning occurred with training on ISO-9, CROSS-9, and CONTROL-9 stimuli, as shown by Figures S1, S2 and S3, which plot the absolute contrast discrimination threshold as a function of the training session for each observer in the three training groups. While the data follow a general trend towards reduction in thresholds with training, thresholds vary considerably between sessions, consistent with previous work (e.g. Beard et al., 1995) [39] as discussed earlier (see “Data analyses” section). Post/pre ratios (PPRs) were calculated for each observer and these values are plotted in Figure 2 for each training group. A PPR of 1.0 indicates no learning and values below 1.0 indicate learning. A repeated measures ANOVA revealed a significant learning effect (F (2, 26) = 4.948, p = 0.02). In each of the three training conditions, the average post-training threshold was reduced from pre-training threshold by a factor of approximately 0.75 to 0.5. Of the three conditions, the highest learning effect was found in the ISO-9 group (post-hoc Tukey’s multiple comparison tests for CROSS-9 vs ISO-9: mean difference −0.22 (p = 0.03), and CONTROL-9 vs ISO-9: mean difference −0.25 (p = 0.02)). However, learning was not significantly different between CROSS-9 and CONTROL-9 groups (mean difference −0.03; p = 0.92).

**Figure 4:**
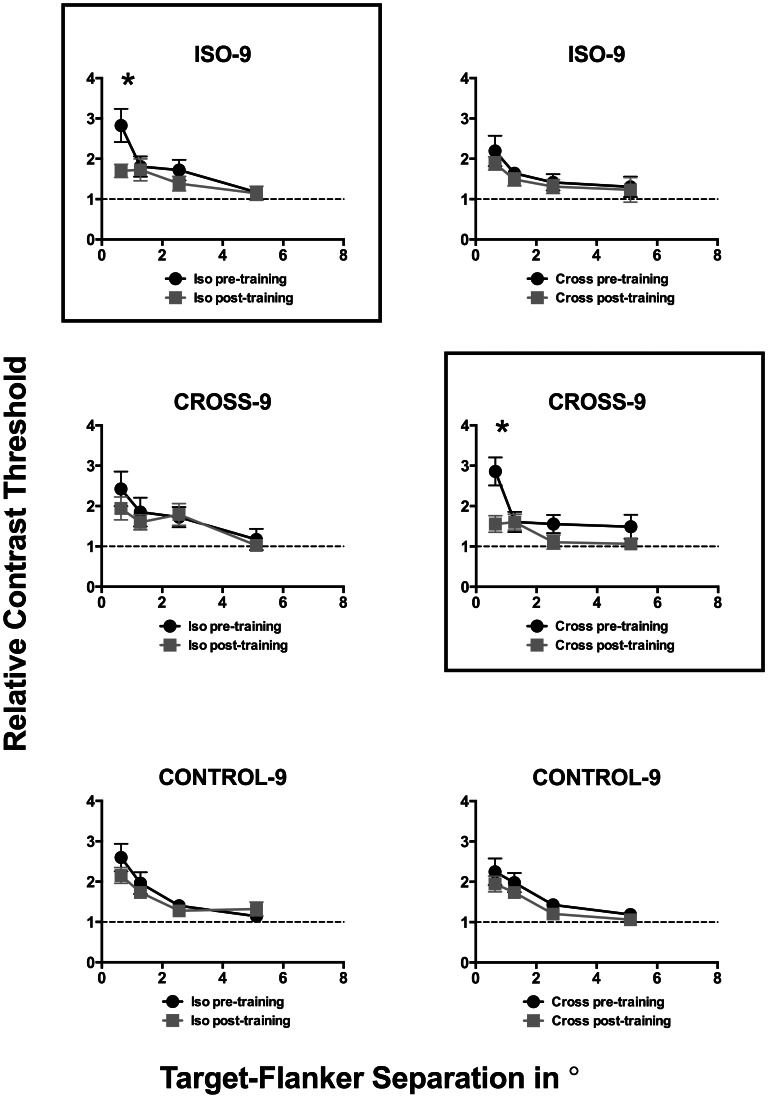
Relative thresholds plotted (in the same format as Figure 3) as a function of target-flanker separation. As in Figure 3, these data are organised to represent the different combinations of training and test stimulus configurations. The horizontal dashed line indicates relative threshold of 1, at which no crowding effect is evident.

Figures 3 and 4 show relative thresholds measured before (black circles) and after (gray squares) training, as a function of eccentricity (Figure 3) and target-flanker separation (Figure 4). A relative threshold of 1 (indicated by the dashed line in each individual plot in Figures 3 and 4) indicates no crowding, with no difference in the absolute CDT between the flanked and unflanked (Control) conditions. One-sample t-tests (corrected for multiple comparisons at an alpha of 0.05) confirmed that significant crowding was found in all conditions at 4.5 and 9 degrees eccentricities in both pre- and post-training phases (p≤0.01). The results are consistent with the well-established finding that the crowding effect is smaller at the fovea than in the periphery (e.g. Leat et al., 1999) [40]. Crowding was also affected by target-flanker separation, with significant crowding found only at small target-flanker separations of 0.64° and 1.28° (p≤0.01).

Repeated measures ANOVA (comparing relative thresholds at each eccentricity and target-flanker separation in pre- and post-training phases) was conducted for different pairings of training and test stimulus configurations (i.e. the different panels in Figures 3 and 4). A main effect of learning was observed (p<0.05) only for the pairings in which the same stimulus parameters were used in training and testing during pre- and post-training phases. Post-hoc Bonferroni tests (corrected for multiple comparisons at a confidence level of 0.05) for these conditions revealed that the pre- and post- training relative thresholds were significantly different (denoted by * in Figures 3 and 4) only at the trained eccentricity of 9 degrees (ISO-Iso – mean difference: 0.86, p = 0.01; CROSS-Cross – mean difference: 0.71, p = 0.01) and target-flanker separations of 0.64 degrees (i.e., ISO-Iso – mean difference: 1.12, p = 0.003; CROSS-Cross – mean difference: 1.3, p = 0.001). These analyses also revealed a main effect for both eccentricity and target-flanker separation (p<0.05) across all different pairings of training-test stimulus configurations. However, no significant interaction effects were observed indicating that changing either eccentricity or target-flanker separation had similar effects on pre- and post- training relative thresholds.

## Discussion

We find learning that is specific to the configuration, eccentricity and target-flanker separation of the trained stimulus. This suggests that the learning is not procedural, related to the subject’s task of discriminating the target from its pedestal, since the task was identical throughout the study. If the improvements in discrimination threshold reflected a general improvement in ability to do this task, or an increased ability to separate target from flankers via attentional mechanisms, those improvements should have also occurred for untrained stimulus conditions. The findings also indicate that the subject learns to the see the whole stimulus (target plus flankers) and not only the target.

Previous studies suggest that perceptual learning that does not transfer to other stimuli reflects changes at an early level of visual processing [32], [41] such as the striate cortex, where cells are tuned to a narrow range of orientations and spatial frequencies (e.g. Tootell et al., 1988) [42]. The training stimuli used in the present study consisted of a target Gabor with or without flanking Gabors, in which all target Gabors had the same orientation and spatial frequency. Therefore, we cannot know whether learning transferred to any untrained target orientations or spatial frequencies. However, we measured thresholds at various target-flanker separations, eccentricities and target-flanker configurations before and after training, and found that learning occurred only in the trained condition and did not transfer to an untrained configuration, location or target-flanker separation.

Training in the present study was performed at 9 degrees and at the smallest possible target-flanker separation, while the pre- and post-training measurements were also taken at other eccentricities and target-flanker separations (see sections 4.1. and 4.2.). The learning is more likely to be location-specific at a low than a high level of visual processing because receptive fields increase in size at higher levels (e.g. Smith et al., 2001) [43]. Thus, the location-specificity of learning found here suggests lower level learning; however we cannot comment on closer eccentricities than 4.5 degrees, since we did not carry out pre- and post-training tests at closer locations.

Our results indicate that the observers’ visual systems learned to discriminate the target embedded within context; contrast discrimination was improved after training, but only for the stimulus configuration used for training. For example, learning with horizontal flankers (ISO) did not improve discrimination of the same target with vertical flankers (CROSS). This finding suggests that training improved processing of the unit combining inputs from the target and flankers, rather than the target per se, and is in agreement with previous work on perceptual learning [30], [35], [36].

Crist et al. (1997) found that perceptual learning on spatial localisation of a bar target presented at 5 degrees from fixation was specific to the configuration of adjacent bars. Maniglia et al. (2011) trained the normal periphery on a contrast detection task with collinear flanking stimuli, and found increased contrast sensitivity for the target with collinear flankers, but not for the same target with orthogonal flankers. Similarly, Hussain et al. (2012) trained the normal periphery on a task involving identification of a letter surrounded (crowded) by other letters. They measured crowded and uncrowded (isolated) letter acuity before and after training, and found that perceptual learning occurs only for flanked letters (the trained condition) and not for isolated (untrained) letters. These findings suggest that learning in detection, localisation or identification tasks, is based on the whole stimulus (context) and not specifically on the target. The present study shows the same type of learning in a contrast discrimination task.

Adini et al. (2002) proposed the idea of context-enabled learning, based on their finding that contrast discrimination learning is facilitated by the presence of flanking stimuli [31]. Yu et al. (2004), however, demonstrated that learning occurs in this type of task without flanking stimuli, and suggested that context-enabled learning is simply learning to see the target and that the context (flankers) have little or no effect [32]. In the present study, a contrast discrimination task of this kind was employed, with training on targets without context (the Control or no-flanker condition) and with context similar to (ISO) or different from (CROSS) the target. We found that learning was greatest when the target and flankers were similar (ISO) but was significant in all three configurations, indicating that perceptual learning occurs both with and without flankers.

Ahissar and Hochstein (1997) found that transfer of perceptual learning occurs in easy but not difficult training tasks [33], with training on a difficult task involving priming at low levels of the visual system, while training on easy tasks can occur without low-level involvement. The task employed in the present study involved simultaneous comparison of two stimuli separated by 18 degrees, one located above and one below fixation, within a 250 ms time period. The difficulty of this task is likely to have been high, since subjects needed to attend to two peripheral locations simultaneously, in which case Ahissar and Hochstein’s theory would suggest low level learning. This possibility is supported by our finding that learning did not transfer to eccentricities or target-flanker separations different from the training condition, since previous studies suggest that learning that is specific to the trained stimulus parameters indicates learning at lower cortical levels [18], [20].

We found that the effect of flankers, referred to here as a crowding effect, is reduced by perceptual learning, and that the reduced crowding occurs only at the trained target-flanker configuration, eccentricity and separation. Reduced crowding in the normal periphery has been demonstrated previously, with training on a contrast detection task [30] or a letter identification task [36]. The present findings indicate that the improvement that occurs in peripheral contrast discrimination is specific to the configuration of the stimulus, including the orientation and location of flankers. It includes not only a reduction in target threshold but also weakening of any inhibitory effect of flankers (crowding). In agreement with previous work on spatial localisation and letter identification [30], [35], [36], the findings suggest that perceptual learning involves learning to see not only the target but also any associated features such as the flanking Gabor stimuli used here.

## Supporting Information

Figure S1 **Contrast discrimination threshold (%) is plotted as a function of training blocks for each observer in ISO-9 group.**
(TIFF)

Figure S2 **Contrast discrimination threshold (%) is plotted as a function of training blocks for each observer in CROSS-9 group.**
(TIFF)

Figure S3 **Contrast discrimination threshold (%) is plotted as a function of training blocks for each observer in CONTROL-9 group.**
(TIFF)
