# Supplementary figures and images for: Context and Crowding in Perceptual Learning on a Peripheral Contrast Discrimination Task: Context-Specificity in Contrast Learning
Source: PLoS One. 2013 May 16;8(5):e63278. doi: 10.1371/journal.pone.0063278 (PMC3655984; doi:10.1371/journal.pone.0063278)

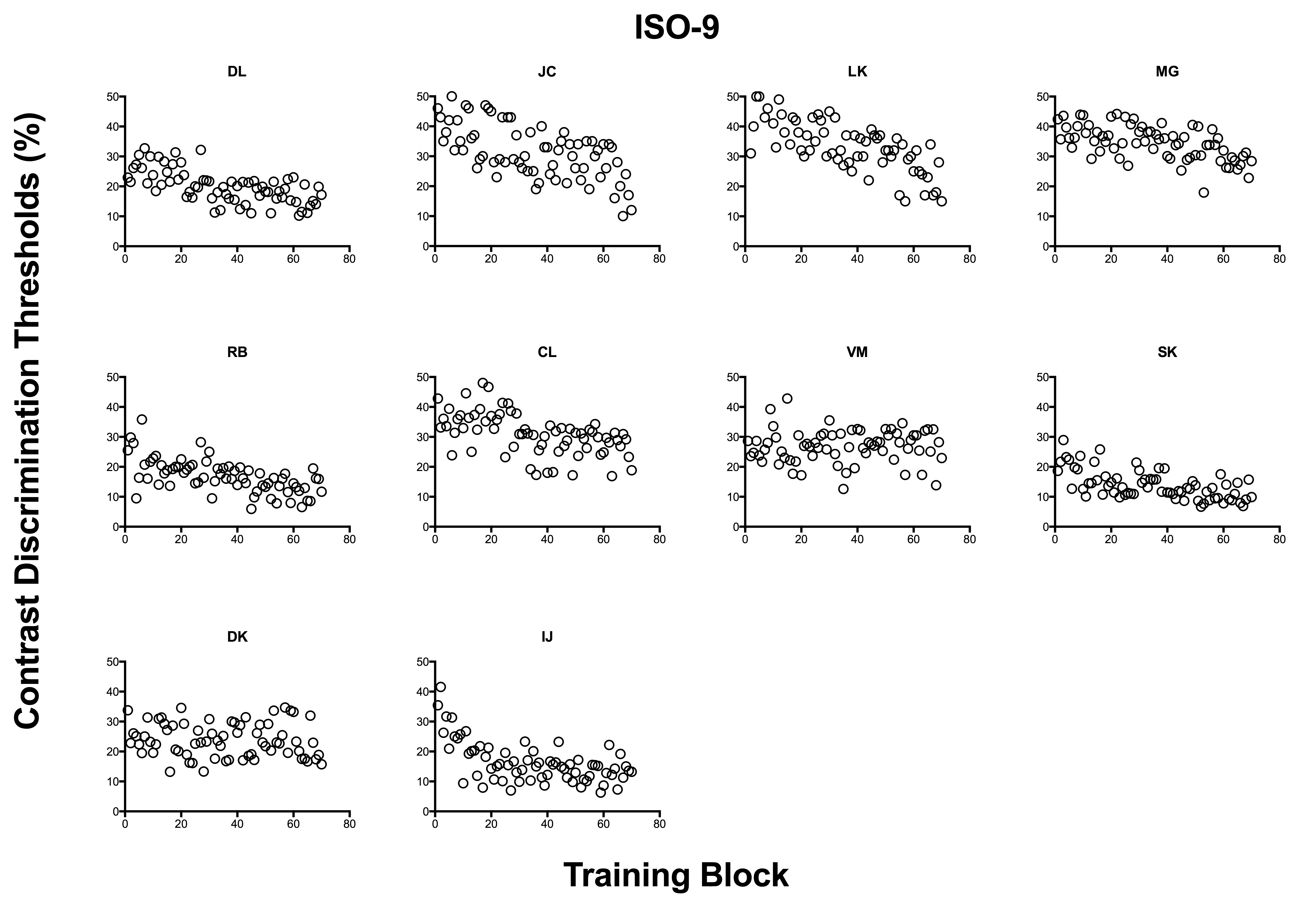

Supplement: Figure S1 — Contrast discrimination threshold (%) is plotted as a function of training blocks for each observer in ISO-9 group. (TIFF) [file pone.0063278.s001.tiff]

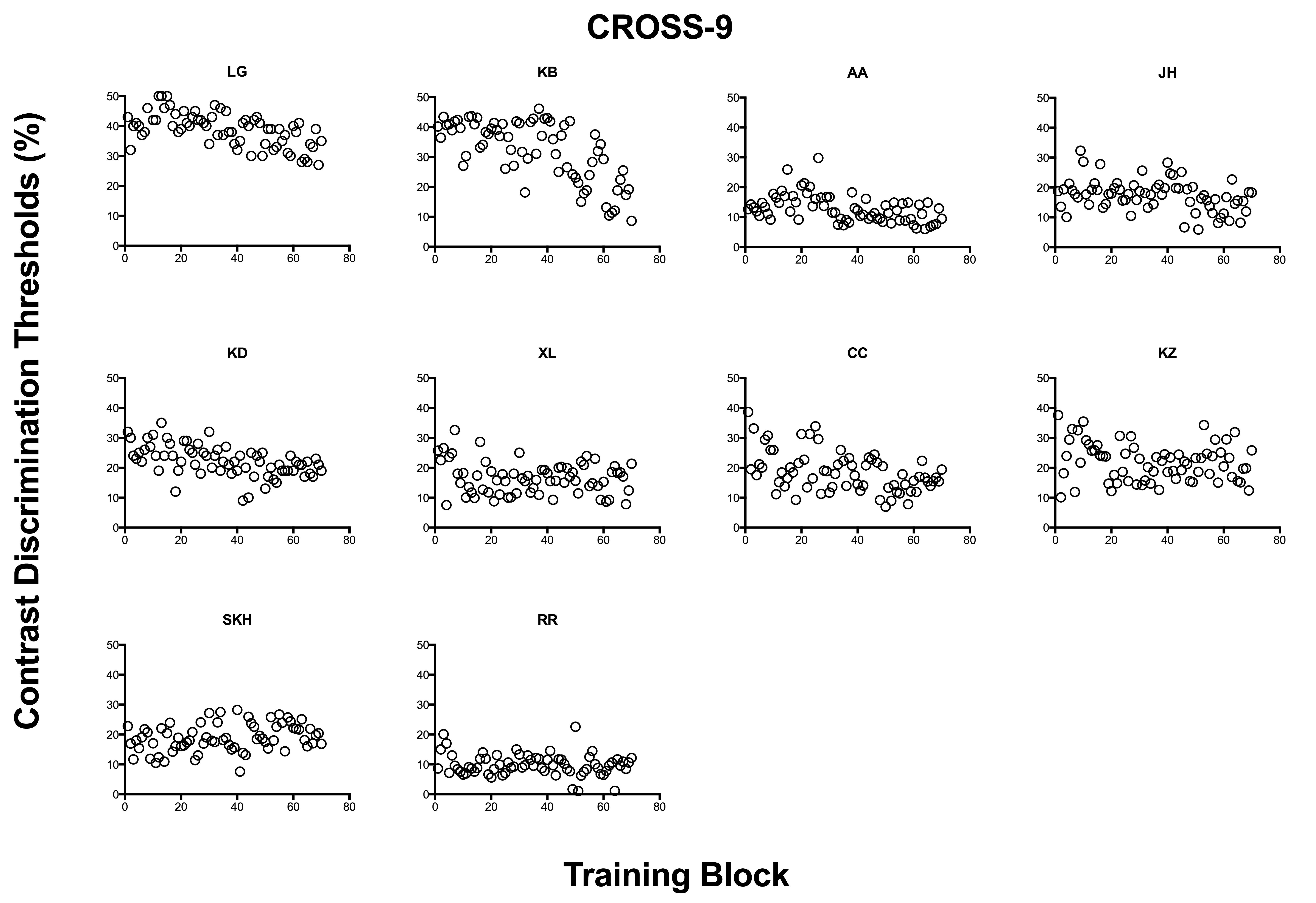

Supplement: Figure S2 — Contrast discrimination threshold (%) is plotted as a function of training blocks for each observer in CROSS-9 group. (TIFF) [file pone.0063278.s002.tiff]

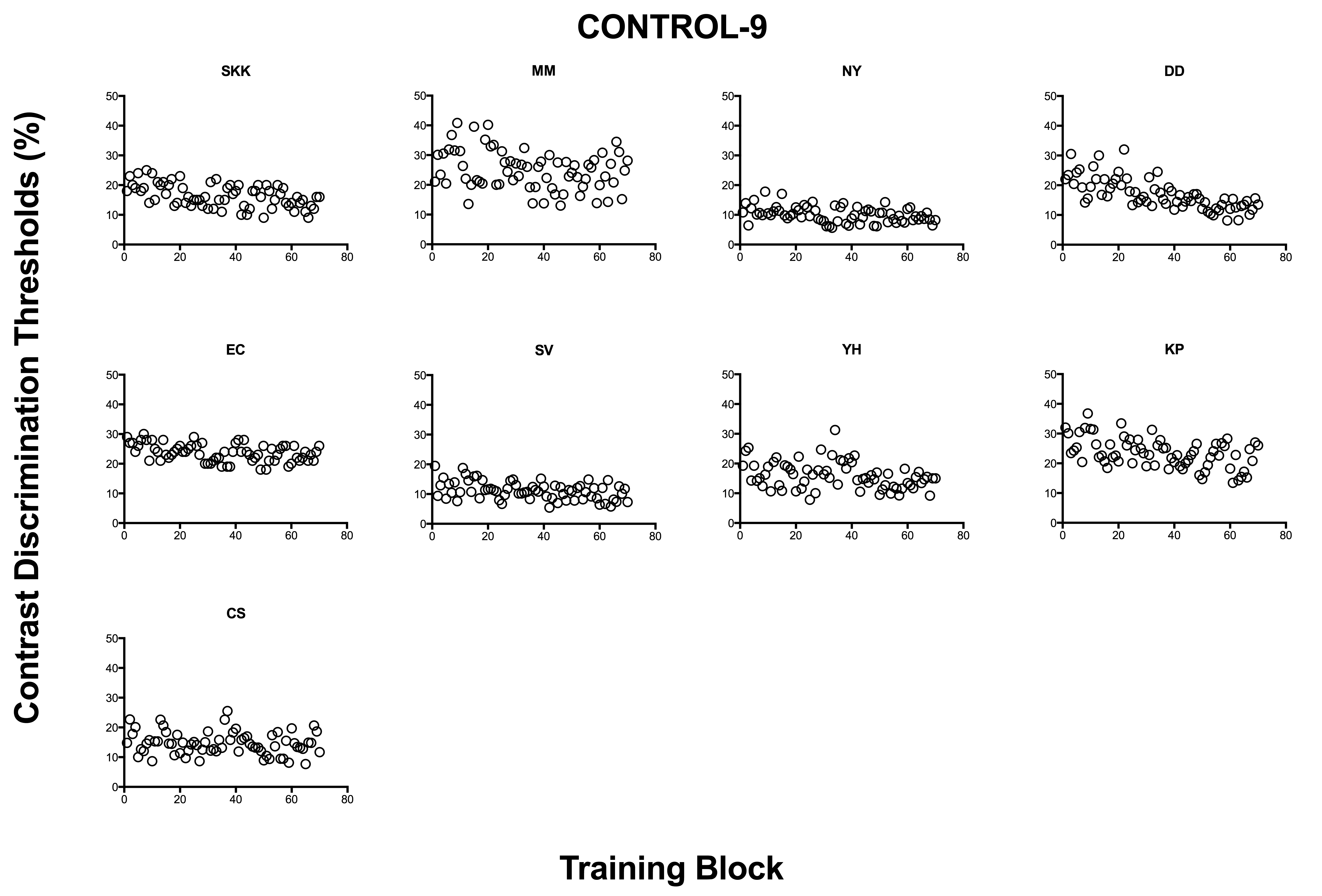

Supplement: Figure S3 — Contrast discrimination threshold (%) is plotted as a function of training blocks for each observer in CONTROL-9 group. (TIFF) [file pone.0063278.s003.tiff]
